# Effect of providing drug utilization review information on tricyclic antidepressant prescription in the elderly

**DOI:** 10.1007/s10916-018-1061-z

**Published:** 2018-09-13

**Authors:** Mi-Ju Park, Mi-Hee Kim, Sun Mi Shin, Soo Youn Chung

**Affiliations:** Korea Institute of Drug Safety and Risk Management, 30, Burim-ro, 169beon-gil, Dongan-gu, Anyang-si, Gyeonggi-do South Korea

**Keywords:** Drug utilization review, Tricyclic antidepressant, Interrupted time series, Elderly, Depression

## Abstract

Tricyclic antidepressants are known as potentially inappropriate medications in the elderly. A notification issued in July 2015 in South Korea recommended caution while prescribing tricyclic antidepressants to the elderly. Further, since October 2015, the nationwide computerized drug utilization review monitoring system provides a pop-up window, on a real-time basis, whenever tricyclic antidepressants are prescribed to elderly outpatients. Therefore, we evaluated whether providing drug utilization review information was effective in reducing tricyclic antidepressant prescription to elderly outpatients. We used the Health Insurance Review and Assessment Service-Adult Patient Sample data from 2014 to 2016. Data related to the prescription of tricyclic antidepressants to outpatients aged 65 years or more were extracted. We determined the number of prescriptions per day per 100,000 elderly patients in each month, compared the average number of prescriptions before and after the drug utilization review information was provided, and evaluated the changes in the number of prescriptions by using an interrupted time series analysis. The average number of tricyclic antidepressant prescriptions per day per 100,000 elderly patients decreased from 76.6 (75.5 to 77.6) to 65.7 (64.5 to 66.9), a 14.2% reduction after the provision of drug utilization review information started. Following initiation of provision of drug utilization review information, there was an immediate drop of 9.2 tricyclic antidepressant prescriptions per day per 100,000 elderly patients, whereas there was no statistically significant change in trends. Providing the drug utilization review information on tricyclic antidepressant prescription for the elderly contributed to the reduction in tricyclic antidepressant prescriptions.

## Introduction

The World Health Organization has reported that depression contributes greatly to the worldwide disease burden: depression was the third leading contributor in 2004 and is predicted to be the leading contributor by 2030 [1]. In South Korea, the number of patients being treated for depression increased by an annual average of 3.1% between 2011 and 2015; specifically, the number of elderly patients aged 65 years or more being treated for depression increased by an annual average of 6.4% during the same period [2].

Tricyclic antidepressants (TCAs) are primarily used as antidepressants; however, several guidelines do not recommend TCA use in the elderly. According to the Beers criteria [3], PRISCUS list [4], Norwegian General Practice (NORGEP) criteria [5], and French consensus panel list [6], TCAs are potentially inappropriate medications (PIMs) for the elderly regardless of the disease, owing to their highly anticholinergic, cognitive impairment, and cardiotoxic effects. According to the Screening Tool of Older People’s Prescriptions and Screening Tool to Alert to Right Treatment (STOPP/START) [7] and McLeod’s criteria [8], TCAs are PIMs for the elderly with specific diseases such as dementia, glaucoma, cardiac conductive abnormalities, and benign prostatic hyperplasia due to the risk of worsening of these conditions.

On July 28, 2015, the Ministry of Food and Drug Safety (MFDS) in South Korea announced a list of medicines that should be used with caution for the elderly aged 65 years or more; the list included all the TCAs used to treat depression in South Korea. Since October 1, 2015, nearly every prescriber who prescribes TCAs to the outpatients aged 65 years or more receives the following real-time pop-up window by a nationwide computerized drug utilization review (DUR) monitoring system [9]: “TCAs should be cautiously administered at low doses to the elderly because of adverse events such as orthostatic hypotension, stagger, dry mouth, dysuria, constipation, and increased intraocular pressure due to an anticholinergic effect.”

However, little is known about the nationwide effect of providing DUR information on drug prescription for the elderly. Therefore, in this study, we evaluated whether provision of DUR information is effective in reducing TCA prescriptions in the elderly.

### Backgrounds in South Korea

In South Korea, DUR information is provided to prescribers and pharmacists via a concurrent DUR system when they prescribe or dispense drugs. Hospital and pharmacy computer systems are linked to the DUR system for real-time transmission of prescription and dispensation information. The DUR information is provided in collaboration with several government organizations [9]. The Korea Institute of Drug Safety and Risk Management (KIDS) developed the DUR information to provide guidance on medications for healthcare professionals. MFDS releases the DUR information to the public after reviewing its credibility. The computerized DUR system is operated by the Health Insurance Review and Assessment Service (HIRA). In South Korea, 99.6% of all medical institution and pharmacies have established DUR services, and 97.2% used the DUR system in August 2017 [10]. Currently, seven types of DUR information are being provided through the above-mentioned process. DUR information related to drug–drug interactions, age restriction, and contraindication in pregnant women require the prescriber to enter a reason for prescribing; if the reason is not entered, the prescriber is reimbursed a reduced medical fee-for-service. Prescribers are not required to manually enter the reasons; they are required only to click on the pop-up window for DUR information related to therapeutic duplication, incorrect dosage, incorrect treatment duration, and PIMs in the elderly.

## Material and methods

### Data source

We used the HIRA-Adult Patient Sample (APS) data for this study [11]. The HIRA-APS data include a sex- and an age-stratified random sample of 20% of the elderly patients aged 65 years or more from the HIRA database. The HIRA database contains National Health Insurance claims data [12], including information on healthcare services, such as diagnoses, procedures, and prescriptions for about 98% of the Korean population, approximately 46 million people as of 2011 [11]. Thus, we could assess the overall use of healthcare service for elderly patients in South Korea by using these nationally representative data.

### Study subjects

We extracted the data for TCAs (amitriptyline, amoxapine, clomipramine, dosulepin, imipramine, nortriptyline, and quinupramine) prescribed to outpatients aged 65 years or more from HIRA-APS. Data of other antidepressants (citalopram, escitalopram, fluoxetine, fluvoxamine, paroxetine, sertraline, duloxetine, milnacipran, venlafaxine, desvenlafaxine, mirtazapine, and bupropion) were also extracted to compare with those of the TCAs. The study period was from January 2014 to December 2016. We included only outpatients in this study because, during the study period, DUR information was provided only for outpatient prescriptions.

### Outcome measure

To evaluate the effect of providing the DUR information for the elderly, we determined the number of outpatient prescriptions per day per 100,000 patients aged 65 years or more in each month by using the following equation:

$$ \frac{\mathrm{Number}\ \mathrm{of}\ \mathrm{outpatient}\ \mathrm{prescriptions}\ \mathrm{for}\ \mathrm{patients}\ \mathrm{aged}\ 65\ \mathrm{years}\ \mathrm{or}\ \mathrm{more}\ \mathrm{in}\ \mathrm{each}\ \mathrm{month}\ }{\mathrm{Number}\ \mathrm{of}\ \mathrm{patients}\ \mathrm{aged}\ 65\ \mathrm{years}\ \mathrm{or}\ \mathrm{more}\ \mathrm{in}\ \mathrm{the}\ \mathrm{year}}\times \frac{\mathrm{100,000}}{\mathrm{Number}\ \mathrm{of}\ \mathrm{days}\ \mathrm{of}\ \mathrm{the}\ \mathrm{month}\ } $$

To adjust variations in day distributions across months, the outcome variable was divided by the number of days of the month. The outcome variable is the number of prescriptions, and not the users, because the pop-up window appears each time a prescription is ordered, so that the change in the number of prescriptions could better reflect the effect of providing the DUR information.

### Statistical analyses

Patient characteristics of the elderly population and TCA users by year were summarized using descriptive statistics. The Charlson comorbidity index (CCI) was determined to evaluate the severity of the disease by using the diagnostic codes for each year [13]. Diseases were diagnosed using the International Classification of Disease, Tenth Revision (ICD-10) code, which has been used in South Korea since 1995 and is constantly being updated [14]. TCA users were classified as patients with depression (ICD-10: F32, F33), dementia (ICD-10: F00, F01, F02, F03, G30, G31, R54, F051), schizophrenia (ICD-10: F20), and other conditions. “Patients with other conditions” refers to patients who had never been diagnosed with depression, dementia, or schizophrenia. The regions in which the facility visited by the patients were located were classified as capital area, big cities in non-capital areas, and rural areas [15]. Patient characteristics by year were compared using the chi-square test.

We calculated the average numbers of TCA prescriptions per day per 100,000 elderly patients before (January 2014 to July 2015) and after (November 2015 to December 2016) the provision of DUR information was started, excluding the period between August 2015 and October 2015 by considering it as a lag period. Although the MFDS announced that TCAs should be used with caution in the elderly aged 65 years or more on July 28, 2015, this information was given to healthcare providers through a nationwide computerized DUR system from October 1, 2015. The average number of other antidepressant prescriptions per day per 100,000 elderly patients was also determined for comparison with the corresponding TCA data. For other antidepressants, the pre-intervention period was from October 2014 to July 2015, excluding the period between January 2014 and September 2014 because of a surge in duloxetine prescriptions until September 2014, due to patent expiration of duloxetine, which accounted for a large portion of antidepressant prescriptions. The lag period and post-intervention period were the same as those for TCAs. The differences between the before and after averages were determined for TCAs and other antidepressants and were evaluated using the two-sample t-test.

To determine the impact of the intervention, a segmented regression analysis with an interrupted time series design was used [16–19]. The pre-intervention, lag, and post-intervention periods were the same as those for the analysis of differences between the average number of prescriptions before and after the provision of DUR information started. The following segmented regression model was used:

$$ {Y}_t={\upbeta}_0+{\upbeta}_1{Time}_t+{\upbeta}_2{Intervention}_t+{\upbeta}_3 Time\ after\ {Intervention}_t+{e}_t $$

Y_t_ is the dependent variable indicating number of prescriptions per day per 100,000 elderly patients in each month at time t. Time is a continuous variable indicating the time in months (a sequential number starting from January 2014 for TCAs and from October 2014 for other antidepressants). Intervention was a dummy variable indicating the time periods in which the intervention was in effect (0 until July 2015 and 1 from November 2015). Time after intervention is a continuous variable indicating time in months after intervention (0 until July 2015, sequential number starting from November 2015). ß_0_ indicates the number of prescriptions per day per 100,000 elderly patients at time 0 (intercept); ß_1_ signifies the secular trend in prescriptions before intervention (baseline trend); ß_2_ denotes the immediate impact after intervention (change in level); and ß_3_ indicates the continuing effect after intervention (change in trend). We determined all coefficients ß_0_–ß_3_ using the maximum likelihood method. Furthermore, we predicted absolute and relative change by comparing estimates using a final regression model, with estimates assuming a continuation of the baseline trend at the end of the study period (December 2016) [20]. When assessing the autocorrelation in the data for the regression model, we used the stepwise method to select the order of the autoregression model. Furthermore, we examined whether the Durbin-Watson statistic was close to 2 to check whether serious autocorrelation remained in the regression model [21, 22]. After adjusting the models for autocorrelation, we confirmed the Durbin-Watson statistics were close to 2 for all the final models.

Regarding the assessment of the impact of providing DUR information by subgroups, we estimated changes in levels and trends for TCA prescription according to characteristics of the patients (sex, age, CCI, and diagnosis of a psychiatric disorder) and the medical institutions (region and type of facility).

We used SAS 9.3 software (SAS Institute Inc., Cary, NC, USA) for all statistical analyses. A two-tailed value of *P* < 0.05 was considered statistically significant. This study was approved by the Institutional Review Board of the Korea Institute of Drug Safety and Risk Management (study ID: KIDS-IRB-2017-7).

## Results

As summarized in Table 1, we identified a total of 1,294,542, 1,276,224, and 1,327,455 patients aged 65 years or more in 2014, 2015, and 2016, respectively. The overall proportion of outpatients who were prescribed TCAs among the elderly population in 2016 decreased compared to that in 2014 (80,217 among 1,294,542, 6.2% and 72,287 among 1,327,455, 5.4% in 2014 and 2016, respectively). TCA users were predominantly female, aged less than 79 years, and diagnosed with depression, as well as had a CCI score of ≥4. Most users were prescribed TCAs in clinics. The most commonly prescribed TCA was amitriptyline.

**Table 1:** Characteristics of the elderly population and tricyclic antidepressant users by year

Characteristics	Elderly population (age ≥ 65 years)							Tricyclic antidepressant users (age ≥ 65 years)						
	2014	(%)	2015	(%)	2016	(%)	*P* value*	2014	(%)	2015	(%)	2016	(%)	*P* value*
Total	1,294,542		1,276,224		1,327,455			80,217		76,466		72,287		
Sex														
Male	539,864	(41.7)	532,015	(41.7)	556,699	(41.9)	<0.001	27,869	(34.7)	26,943	(35.2)	25,648	(35.5)	<0.001
Female	754,678	(58.3)	744,209	(58.3)	770,756	(58.1)		52,348	(65.3)	49,523	(64.8)	46,639	(64.5)	
Age														
(Mean ± SD)	73.9 ± 6.8		74.6 ± 6.6		74.7 ± 6.7			74.1 ± 6.2		74.7 ± 6.1		74.8 ± 6.2		
65-69	404,498	(31.2)	354,633	(27.8)	371,056	(28.0)	<0.001	21,059	(26.3)	18,011	(23.6)	17,716	(24.5)	<0.001
70-74	357,653	(27.6)	356,459	(27.9)	353,835	(26.7)		23,748	(29.6)	22,170	(29.0)	19,829	(27.4)	
75-79	270,143	(20.9)	279,214	(21.9)	292,659	(22.0)		19,848	(24.7)	19,638	(25.7)	18,257	(25.3)	
80-84	156,022	(12.1)	171,233	(13.4)	186,293	(14.0)		10,577	(13.2)	11,168	(14.6)	11,238	(15.5)	
≥ 85	106,226	(8.2)	114,685	(9.0)	123,612	(9.3)		4985	(6.2)	5479	(7.2)	5247	(7.3)	
Charlson comorbidity index														
(Median, IQR)	(2, 1 to 3)		(2, 1 to 4)		(2, 1 to 4)			(3, 2 to 5)		(3, 2 to 5)		(3, 2 to 5)		
0	270,780	(20.9)	249,107	(19.5)	245,684	(18.5)	<0.001	6223	(7.8)	5649	(7.4)	4948	(6.8)	<0.001
1	291,211	(22.5)	277,176	(21.7)	280,121	(21.1)		12,311	(15.3)	11,210	(14.7)	9880	(13.7)	
2	242,484	(18.7)	237,970	(18.6)	247,052	(18.6)		14,682	(18.3)	13,314	(17.4)	12,399	(17.2)	
3	175,470	(13.6)	177,891	(13.9)	186,382	(14.0)		13,566	(16.9)	12,612	(16.5)	11,878	(16.4)	
4+	314,597	(24.3)	334,080	(26.2)	368,216	(27.7)		33,435	(41.7)	33,681	(44.0)	33,182	(45.9)	
Diagnosis of a psychiatric disorder														
Depression	164,299	(12.7)	172,822	(13.5)	182,047	(13.7)	<0.001	44,671	(55.7)	44,098	(57.7)	42,877	(59.3)	<0.001
Dementia	169,494	(13.1)	189,340	(14.8)	219,167	(16.5)	<0.001	14,870	(18.5)	15,900	(20.8)	16,487	(22.8)	<0.001
Schizophrenia	9921	(0.8)	11,286	(0.9)	11,848	(0.9)	<0.001	716	(0.9)	825	(1.1)	858	(1.2)	<0.001
Others	1,009,536	(78.0)	970,006	(76.0)	990,358	(74.6)	<0.001	30,848	(38.5)	27,511	(36.0)	24,507	(33.9)	<0.001
Region														
Capital area	638,928	(49.4)	635,126	(49.8)	666,688	(50.2)	<0.001	29,288	(36.5)	27,475	(35.9)	26,499	(36.7)	0.008
Big cities in non-capital areas	343,750	(26.6)	342,093	(26.8)	358,765	(27.0)	<0.001	18,245	(22.7)	17,281	(22.6)	16,643	(23.0)	0.142
Rural areas	550,586	(42.5)	546,824	(42.8)	562,149	(42.3)	<0.001	33,692	(42.0)	32,597	(42.6)	29,956	(41.4)	<0.001
Type of facility														
Tertiary	708,788	(54.8)	717,493	(56.2)	767,108	(57.8)	<0.001	28,998	(36.1)	27,636	(36.1)	28,088	(38.9)	<0.001
Secondary	477,337	(36.9)	490,465	(38.4)	520,049	(39.2)	<0.001	8801	(11.0)	8488	(11.1)	7838	(10.8)	0.283
Clinics	1,219,990	(94.2)	1,206,194	(94.5)	1,254,075	(94.5)	<0.001	46,048	(57.4)	43,785	(57.3)	39,422	(54.5)	<0.001
Tricyclic antidepressant prescribed														
Amitriptyline								58,192	(72.5)	54,216	(70.9)	50,079	(69.3)	<0.001
Nortriptyline								19,565	(24.4)	19,543	(25.6)	19,736	(27.3)	<0.001
Imipramine								5590	(7.0)	5412	(7.1)	4945	(6.8)	0.200
Others								252	(0.3)	240	(0.3)	213	(0.3)	0.740

*P value determined using the chi-square test

SD: Standard deviation, IQR: interquartile range

**Fig. 1:**
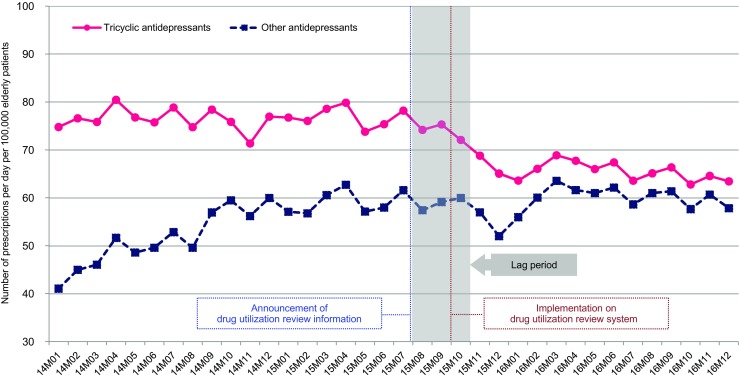
Number of prescriptions per day per 100,000 elderly patients in each month for tricyclic antidepressants and other antidepressants

Figure 1 shows the monthly prescription trend for TCAs and other antidepressants. TCA prescriptions continued to decline after the provision of DUR information was started until January 2016; thereafter, it remained relatively steady until the end of 2016. Prescriptions for other antidepressants increased rapidly until October 2014 as a patent on duloxetine expired in August 2014. No notable changes were observed in the trend of prescriptions for other antidepressants compared to that for TCAs after the provision of DUR information was initiated.

Before the provision of DUR information started, the average number of TCA prescriptions per day per 100,000 elderly patients was 76.6 (95% confidence interval [CI]: 75.5 to 77.6). After the provision of DUR information started, the average number of TCA prescriptions per day per 100,000 elderly patients was 65.7 (95% CI: 64.5 to 66.9). The difference between in the average numbers before and after the provision of DUR information started was −10.9 (95% CI: -12.4 to −9.4) for TCAs, resulting in a 14.2% relative reduction. However, there was no significant difference for other antidepressants (Table 2).

**Table 2:** Average numbers of prescriptions per day per 100,000 elderly patients before and after the provision of drug utilization review information started

	Average number of prescriptions per day per 100,000 elderly patients			
	Before providing DUR information (95% CI)	After providing DUR information (95% CI)	Difference (95% CI)	*P* value*
Tricyclic antidepressants†	76.6 (75.5 to 77.6)	65.7 (64.5 to 66.9)	−10.9 (−12.4 to −9.4)	<0.001
Other antidepressants‡	59.0 (57.3 to 60.7)	59.4 (57.6 to 61.2)	0.4 (−2.0 to 2.7)	0.733

* P value determined by the two-sample t-test

† For tricyclic antidepressants, the pre-intervention period was from January 2014 to July 2015, and the post-intervention period was from November 2015 to December 2016

‡ For other antidepressants, the pre-intervention period was from October 2014 to July 2015 excluding the period between January 2014 and September 2014 because of a surge in duloxetine prescriptions until September 2014, due to patent expiration of duloxetine. The post-intervention period was from November 2015 to December 2016

DUR: Drug utilization review, CI: Confidence interval

Table 3 shows estimates determined using the segmented regression analyses. After the provision of DUR information started, there was an immediate drop of 9.2 TCA prescriptions per day per 100,000 elderly patients (*P* < 0.001), but there were no statistically significant changes in trends (*P* = 0.135). This decline was expected to result in a reduction of 12.6 (95% CI: -17.1 to −8.1) TCA prescriptions per day per 100,000 elderly patients on a monthly basis in December 2016, equating to an approximate reduction of 16.5% (95% CI: -21.6% to −11.3%). There was a significant reduction in TCA prescriptions for the elderly patients with a CCI of 0 in terms of both level and trend, with the predicted reduction being 6.3 (95% CI: -8.1 to −4.4) prescriptions per day per 100,000 elderly patients and an approximately 21.1% (95% CI: -26.5% to −15.8%) reduction in December 2016. TCA prescriptions in rural areas and clinics also showed a significant reduction in terms of both level and trend. This decline would be expected to result in 16.9 (95% CI: -21.5 to −12.3) and 9.8 (95% CI: -12.7 to −6.9) reduction in number of TCA prescriptions per day per 100,000 elderly patients in each month, respectively, equating to approximately 20.0% (95% CI: -24.7% to −15.4%) and 19.3% (95% CI: -24.2% to −14.4%) reduction for both in December 2016. No significant change in level or trend was observed for other antidepressants.

**Table 3:** Segmented regression model estimates for number of prescriptions per day per 100,000 elderly patients in each month

		Intercept		Baseline trend		Change in level		Change in trend		Predicted absolute change		Predicted relative change (%)	
		Estimate	*P* value	Estimate	*P* value	Estimate	*P* value	Estimate	*P* value	Estimate	95% CI	Estimate	95% CI
Tricyclic antidepressants*		76.50	<0.001	0.008	0.929	−9.15	<0.001	−0.25	0.135	−12.6	(−17.1, −8.1)	−16.5	(−21.6, −11.3)
	Sex												
	Male	62.85	<0.001	0.12	0.106	−8.17	<0.001	−0.24	0.077	−11.6	(−15.3, −7.9)	−17.4	(−22.2, −12.6)
	Female	86.27	<0.001	−0.07	0.490	−9.81	<0.001	−0.25	0.205	−13.3	(−18.6, −8.0)	−15.8	(−21.4, −10.3)
	Age												
	65-79 years	74.56	<0.001	−0.03	0.756	−8.57	<0.001	−0.26	0.110	−12.2	(−16.6, −7.8)	−16.6	(−21.7, −11.4)
	≥ 80 years	84.61	<0.001	0.05	0.671	−11.30	<0.001	−0.16	0.472	−13.5	(−19.4, −7.5)	−15.6	(−21.7, −9.6)
	Charlson comorbidity index												
	0	26.91	<0.001	0.08	0.034	−3.31	<0.001	−0.21	0.004	−6.3	(−8.1, −4.4)	−21.1	(−26.5, −15.8)
	1	52.35	<0.001	−0.10	0.038	−7.19	<0.001	0.00	0.963	−7.2	(−9.6, −4.9)	−14.7	(−18.8, −10.7)
	2	73.33	<0.001	−0.12	0.234	−7.48	0.000	−0.16	0.401	−9.7	(−14.8, −4.6)	−14.0	(−20.5, −7.4)
	3	96.66	<0.001	−0.16	0.062	−15.42	<0.001	0.06	0.692	−14.6	(−18.9, −10.3)	−15.9	(−19.9, −12.0)
	4+	135.88	<0.001	−0.36	0.036	−15.74	<0.001	−0.31	0.320	−20.1	(−28.6, −11.6)	−16.2	(−22.2, −10.2)
	Diagnosis of a psychiatric disorder												
	Depression	349.65	<0.001	−0.69	0.114	−33.40	<0.001	−0.36	0.654	−38.4	(−60.5, −16.4)	−11.8	(−17.9, −5.6)
	Dementia	116.93	<0.001	−0.09	0.579	−13.40	<0.001	−0.59	0.052	−21.6	(−30.0, −13.3)	−19.0	(−24.8, −13.1)
	Schizophrenia	84.97	<0.001	0.60	0.037	−5.68	0.309	−0.39	0.453	−11.1	(−26.2, 4.0)	−10.6	(−22.1, 0.9)
	Others	36.41	<0.001	−0.12	0.008	−5.23	<0.001	0.009	0.913	−5.1	(−7.3, −2.9)	−15.7	(−21.8, −9.7)
	Region												
	Capital area	50.84	<0.001	−0.11	0.123	−5.02	0.000	−0.07	0.580	−6.0	(−9.4, −2.5)	−12.6	(−19.2, −6.1)
	Big cities in non-capital areas	65.96	<0.001	−0.14	0.122	−6.48	0.000	0.00	0.984	−6.4	(−10.9, −1.9)	−10.5	(−17.2, −3.7)
	Rural areas	80.23	<0.001	0.12	0.187	−11.40	<0.001	−0.39	0.025	−16.9	(−21.5, −12.3)	−20.0	(−24.7, −15.4)
	Type of facility												
	Tertiary	37.73	<0.001	−0.02	0.818	−5.42	0.001	0.14	0.417	−3.5	(−8.1, 1.1)	−9.4	(−21.0, 2.2)
	Secondary	19.63	<0.001	−0.06	0.061	−1.26	0.020	−0.11	0.062	−2.8	(−4.3, −1.2)	−15.7	(−23.4, −8.0)
	Clinics	51.96	<0.001	−0.03	0.556	−6.23	<0.001	−0.26	0.021	−9.8	(−12.7, −6.9)	−19.3	(−24.2, −14.4)
Other antidepressants†		57.73	<0.001	0.23	0.446	−2.50	0.270	0.01	0.972	−2.3	(−23.9, 19.2)	−3.6	(−19.9, 12.8)

* For tricyclic antidepressants, the pre-intervention period was from January 2014 to July 2015, and the post-intervention period was from November 2015 to December 2016

† For other antidepressants, the pre-intervention period was from October 2014 to July 2015 excluding the period between January 2014 and September 2014 because of a surge in duloxetine prescriptions until September 2014, due to patent expiration of duloxetine. The post-intervention period was from November 2015 to December 2016

CI: Confidence interval

## Discussion

The average number of TCA prescriptions in the elderly decreased by 14.2% after the provision of DUR information started, and there was an immediate drop of 9.2 TCA prescriptions per day per 100,000 elderly patients, whereas there was no statistically significant change in trends. On the other hand, neither the level nor the trend change for other antidepressant prescriptions was statistically significant. This finding confirms that the decline in TCA prescription was not due to a reduction in depression or other policy effects. Furthermore, TCAs appear to have not been replaced by other antidepressants, although selective serotonin reuptake inhibitors (SSRIs), serotonin-norepinephrine reuptake inhibitors (SNRIs), mirtazapine, and bupropion have been proposed as TCA alternatives for depressed elderly [8, 23, 24, 6, 4, 25].

The sub-group analysis showed that the reduction in the levels and trends was significant in patients receiving TCAs with a CCI of 0 and in patients from rural areas and clinics. The predicted relative change rate also decreased more in patients receiving TCAs with a CCI of 0 and in patients from rural areas and clinics, which means that TCA prescription decreased more than that in the other groups, despite considering the fact that the higher the frequency of TCA prescription, the more it decreases. Patients with a severe disease usually choose high-end hospitals in larger cities [26, 27], whereas those with a mild disease tend to be treated more in rural areas and clinics. Medication changes are relatively easy for low-risk patients because of a wide range of drug choices.

The findings of other studies on PIM use in the elderly are consistent with our findings. In ambulatory care clinics of a tertiary medical center, age-specific medication alert messages during computerized provider order entry (CPOE) decreased the incidence of the top 10 most frequently prescribed PIMs including TCAs from 9.0 to 8.3% in the elderly, resulting in a 7.8% reduction [28]. In the elderly admitted to an academic medical center, the prescription of 16 PIMs, including amitriptyline, decreased from 11.56 to 9.94 orders per day, resulting in a 14.0% reduction after introducing the CPOE drug warning system [29].

To our knowledge, no study has analyzed the nationwide effect of providing DUR information for the elderly on drug prescription; on the other hand, studies evaluating the nationwide effect of providing DUR information on contraindicated drugs have been actively performed. Kim et al. [30] reported that the proportion of contraindicated drug-drug interactions between prescriptions decreased from 0.9746 to 0.7944% (*p* = 0.0026) after implementation of the DUR information system. Song et al. [31] conducted a study on changes in the use of age-contraindicated drugs and found an 85.71% (95% CI: 71.53 to 102.72%) reduction in related prescriptions. Providing DUR information on contraindicated drugs during pregnancy resulted in 27.77% (95% CI: 27.64 to 27.90%) reduction in the prescription of these drugs [32].

The effect of providing DUR information on TCA prescription in the elderly was not marked compared to the effect of providing DUR information on contraindicated drugs. The main reason is that prescribers are not required to enter a reason for TCA prescription to the elderly, unlike in the case of contraindicated drugs. To prescribe contraindicated drugs, prescribers are required to enter a reason for prescribing; if the reason is not entered, the prescriber is reimbursed a reduced medical fee-for-service. Second, TCAs seem to be preferred to both prescribers and patients, because they are inexpensive and have been in use for a long time. The third reason is that TCAs can be considered for elderly depression that does not respond to other treatments such as SSRIs or SNRIs according to the guideline [25]. Also, TCAs can be used for various off-label indications including neuropathic pain [33], sleep disorder [34, 35], and headache [36] according to the guideline.

A variety of off-label indications may also be the reason why TCAs have not been replaced by other antidepressants. According to Hwang et al. [37], 20.7% of TCAs were used to treat depression, and 61.5% were used to treat pain. Therefore, TCAs may have been replaced by pain relievers other than non-TCA antidepressants. In addition, TCAs may be used for mild depression or pain because of their low price, and then TCAs may be discontinued or replaced due to concerns regarding side effects after providing the DUR information.

On July 28, 2015, long-acting benzodiazepines have also been announced as PIMs in the elderly with TCAs, and the pop-up window by the DUR system is also being offered to prescribers who prescribe benzodiazepines. Additionally other drugs will continue to be added to the list of medicines that should be used with caution among the elderly aged 65 years or more. In the future, we also need to assess the effect of providing DUR information on benzodiazepines and newly added medicines to compare with TCAs.

This study has several strengths. To our knowledge, this is the first study to evaluate the nationwide effect of providing DUR information for the elderly. Most studies on PIM use in the elderly were conducted in a single hospital unit. Second, various subgroup analyses revealed factors that affect compliance. Compliance was relatively good in patients with a CCI of 0 and in patients from rural areas and clinics, indicating patients with mild disease. A third strength is that the changes were quantified by level and trend separately, and not only by a simple comparison of the before and after averages. A change in the level indicates an immediate result of the policy and the change in the trend indicates a sustained effect. Finally, we compared TCAs with other antidepressants, which showed more clearly the effect of providing DUR information. Prescriptions for both TCAs and other antidepressants decreased immediately after the DUR information on TCA prescription was provided. However, only prescriptions of TCAs remained reduced and prescriptions of other antidepressants returned to their original trend quickly.

There are also several limitations to this study. First, this study evaluated only the influence of providing DUR information in reducing TCA prescriptions in the elderly. Medication changes or changes in health outcomes could not be studied, because HIRA-APS data were extracted yearly and a longitudinal follow-up of individual patients was not possible. Further study using longitudinal data is required. Second, we used diagnostic codes when categorizing patients. Diagnostic codes in claims data should be interpreted with caution, because they are collected mainly for reimbursing healthcare services and not for clinical use. In a previous study [38], the diagnostic consistency rate between medical records and insurance claims for outpatients was 86.1%. Finally, there may have been other interventions that could have affected TCA prescriptions, for example, changes in the criteria for depression and changes in large CPOE systems. However, as shown in Fig. 1, there was no significant change in TCA prescriptions at other time points except immediately after the DUR information was provided.

In conclusion, providing DUR information for the elderly aged 65 years or more had an effect of reducing TCA prescription. The overall TCA prescription decreased immediately, but the trend change was not statistically significant. However, there was a statistically significant decrease in the level and trend in patients receiving TCAs with CCI of 0 and in patients from rural areas and clinics.

## Abbreviations

TCA: Tricyclic antidepressant
NORGEP: Norwegian General Practice
PIM: Potentially inappropriate medication
STOPP/ START: Screening Tool of Older People’s Prescriptions and Screening Tool to Alert to Right Treatment
MFDS: Ministry of Food and Drug Safety
DUR: Drug utilization review
KIDS: Korea Institute of Drug Safety and Risk Management
HIRA: Health Insurance Review and Assessment Service
APS: Adult Patient Sample
CCI: Charlson comorbidity index
ICD-10: International Classification of Disease, Tenth Revision
CI: Confidence interval
SSRI: Selective serotonin reuptake inhibitor
SNRI: Serotonin-norepinephrine reuptake inhibitor
CPOE: Computerized provider order entry
